# Risk of genitourinary late effects after radiotherapy for prostate cancer associated with early changes in bladder shape

**DOI:** 10.1016/j.phro.2025.100855

**Published:** 2025-10-31

**Authors:** Oscar Casares-Magaz, Renata G. Raidou, Katarina Furmanová, Niclas Pettersson, Vitali Moiseenko, John Einck, Austin Hopper, Rick Knopp, Ludvig P. Muren

**Affiliations:** a Danish Centre for Particle Therapy, Aarhus University Hospital, Aarhus, Denmark; b TU Wien, Institute of Visual Computing & Human-Centered Technology, Wien, Austria; c Masaryk University, Faculty of Informatics, Brno, Czech Republic; d Department of Medical Radiation Sciences, Institute of Clinical Sciences, Sahlgrenska Academy, University of Gothenburg, Gothenburg, Sweden; e University of California San Diego, Radiation Medicine and Applied Sciences, San Diego, USA; f University of Kansas Hospital, Department of Radiation Oncology, KS, USA; g Dept of Clinical Medicine, Aarhus University, Aarhus, Denmark

**Keywords:** Prostate cancer radiotherapy, Machine learning, Radiation-induced late effects, Adaptive radiotherapy

## Abstract

**Background and purpose:**

The risk of genitourinary late effects is a major dose-limiting factor in radiotherapy for prostate cancer. By using shape analysis and machine learning, the aim of this study was to evaluate whether bladder shape descriptors from the first week of treatment could identify patients experiencing genitourinary late effects.

**Material and methods:**

From a cohort of 258 prostate cancer patients treated with daily cone-beam computed tomography (CBCT)-guided radiotherapy (prescription doses of 77.4–81.0 Gy), 7 pre-treatment asymptomatic cases experiencing RTOG genitourinary late effects ≥Grade 2 and 21 matched controls were selected. The bladder was manually contoured on each CBCT, and a 17-D vector comprising shape descriptors was used for patient clustering, focusing on bladder contours from the first week of treatment. ANOVA was used to test statistical significance of descriptors across and within clusters.

**Results:**

Of the contours from the first week of treatment, 84 % could be classified in two main clusters with distinct bladder shape characteristics. This cluster stratification remained identical when bladder contours from the entire course of treatment were used. Convexity, elliptic variance and compactness were significantly different between patients with vs. without genitourinary late effects ≥Grade 2 (*p* < 0.05). Dice Coefficients between predictive models using descriptors of the first week and the voxels’ probability of belonging to the bladder were above 93 ± 6 % (median ± interquartile range).

**Conclusion:**

Bladder shape descriptors in the first week of treatment showed potential to predict the risk of developing genitourinary late effects after radiotherapy for prostate cancer.

## Highlights

• Changes in bladder shape during first week are representative of the entire course.
• Bladder shape infers risk of urinary late effects in radiotherapy for prostate cancer.
• Monitoring bladder shape descriptors might enable early treatment adaptation.

## Graphical abstract

**Figure:**
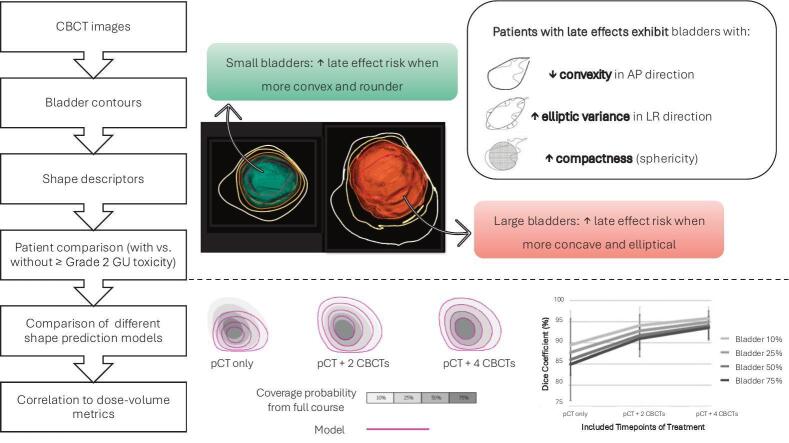

## 1 Introduction

Modern radiotherapy (RT) protocols for prostate cancer allow dose escalation to the prostate, achieving improved clinical outcomes [[1], [2], [3]]. However, genitourinary (GU) late effects are still observed at non-negligible rates [4,5], and bladder doses are thus considered dose-limiting during treatment planning [6]. Associations between GU late effects following RT for prostate cancer and dose/volume parameters in the bladder have been extensively studied for a wide range of treatment techniques and fractionation schedules [6,7], but these associations remain not fully understood. The weak associations observed between dose-volume metrics and occurrence of late effects might be due to considerable changes occurring in bladder volume, shape, and position during the treatment course; where the actually delivered dose tends to significantly differ from the initially planned dose [[8], [9], [10]]. Additionally, planned dose-volume metrics have shown limited predictive power [[10], [11], [12]].

The introduction of image-guided RT enabled studies of inter-fractional changes of bladder volume and spatial dose deposition within the bladder. Implementation of image-guided RT also supported adherence to strict bladder filling protocols, aimed at matching patient’s anatomy when treating to the anatomy seen in the planning computed tomography (CT), thereby reducing differences between planned and delivered dose distributions within target volumes and organs at risk. Additionally, image-guided RT opened for retrospective studies of the impact of the spatial distribution of delivered doses within the bladder, and associated dose–response relationships. It has been shown that increased doses delivered at the base of the bladder, the urethra, and the trigone were associated with a higher risk of developing GU late effects [[13], [14], [15]]. The goal of previous studies was to decipher dose–response relationships allowing the inclusion of valid metrics at the planning stage to accurately predict the risk of late effects.

In this study, we proposed to evaluate patient-specific risk of GU late effects by analyzing planned dose and bladder changes occurring during the first week of the treatment course. By using well-established methods for shape analysis and machine learning algorithms for dimensionality reduction and clustering, the aim of the study was to evaluate whether parameterized shape descriptors of the bladder from the first week of treatment might classify patients with and without exhibiting GU late ≥Grade 2 effects. Additionally, predictive models build from bladder contours from the first week of treatment were evaluated against the actual probability of belonging to the bladder.

## 2 Materials and methods

### 2.1 Patient data and selection of case-control design

A matched case-control study was performed within a total cohort of 258 prostate cancer patients. All patients were treated with external beam RT for prostate cancer at the University of California San Diego, between 2008 and 2014. Prescription doses of 77.4–81.0 Gy in 43–45 fractions were delivered to the intact prostate using daily cone-beam CT (CBCT)-guidance for patient set-up (on fiducial markers implanted prior to treatment) and for adhesion to organ filling protocol: empty rectum and full bladder. This study and the data extraction were approved by the institutional review board of the University of California, San Diego prior to data collection.

Twenty-seven patients (11 %) presented RTOG GU late effects ≥Grade 2. Out of these 27 patients, eight patients (3 %) did not have any symptoms prior to treatment and were considered as cases in this study. This selection criterion for cases was established to evaluate exclusively clear new onset Grade 2 or higher GU score late effects. Each case was matched with three controls based on pretreatment GU symptoms, age, Gleason score, follow-up time, and use of hormone therapy (the potential controls were the remaining patients presenting GU late Grade 0 effects). For one of the cases, it was not possible to find matched controls fulfilling the matching criteria, and the analysis was thus performed for a total of 28 patients, including 7 cases and 3 controls per case; the case without any matched control was excluded [12].

### 2.2 Descriptive analysis

For each of the 28 analyzed patients, 12 CBCTs (daily CBCTs for first week of treatment, and then one weekly CBCT) were rigidly registered to the planning CT using the recorded treatment shifts, and the bladder was manually contoured on each CBCT. Bladder contours were finalized and approved by the responsible radiation oncologist.

A 17-D shape descriptor vector was subsequently computed for each bladder contour including the following shape descriptors: the position of the center of mass (x,y,z) of the bladder referred to treatment isocenter – center of target volume – (n = 3), the orientation of the three cartesian axes (n = 3), the convexity (n = 3), variance (n = 3), and elliptical variance (n = 3) — each measured along the three principal axes — as well as the compactness (n = 1), and the bladder volume (n = 1) [16] (Suppl. Mat. A). These descriptors underwent scaling in the form of normalization (scaling within a given range to ensure their units were comparable) and standardization (centering around (μ,σ)=(0,1)). In this way, each bladder shape, size, and position at each CBCT was described by a vector of 17 comparable values. In order to detect similarities across patients, the 17-D vectors of the bladder at all CBCTs were used as input to a t-distributed Stochastic Neighborhood Embedding (t-SNE) [17] for dimensionality reduction. The output of t-SNE was a reduced 2D vector per bladder, this dimensionality reduction avoided computational load and the need of multiple comparisons correction. Then, a Gaussian Mean-Shift clustering [18] was performed on the 2D vectors provided as the t-SNE outcome, to determine patients with similar anatomies, by grouping them into clusters. Clustering was done based on the first time-point, and cluster matching was performed to ensure correspondence. The outcome of the clustering was a cluster assignment, for each patient at each CBCT.

For the statistical analysis of the resulting clusters (both across clusters and within clusters), ANOVA and t-tests [19] were performed to examine statistically significant differences between clusters. This was done by accounting for all 17 shape descriptors for the planning CT and the first four days, and subsequently for all CBCTs. The analysis included a t-SNE non-linear dimensionality reduction technique, followed by the Gaussian Mean Shift clustering, allowing the ANOVA analysis on shape descriptors across clusters [20] (Suppl. Mat. B). Prior to data analysis, the Shapiro-Wilk normality test was used to test data distribution against normality (significance level p < 0.05).

### 2.3 Generation of bladder shape predictive models

In order to test whether a patient could be classified early in a particular patient cluster, different models for shape prediction based on descriptors from the first week of treatment were generated and tested against data from the entire RT course. By using principal component analysis three predictive models [20,21] were generated for each patient by using the ten closest patients and including incrementally more CBCTs on each: contours from the planning CT **(Model 1)**, contours from the planning CT and the two first CBCTs **(Model 2)**, and contours from the planning CT and the four first CBCTs **(Model 3)**.

Using the bladder contours from the planning CT and the 12 CBCTs of the entire RT course, the bladder volumes for each patient at each CBCT were transformed into a unified volumetric representation by creating binary masks and rigidly registered CBCTs at the treatment isocenter (using the fraction registration file generated with patient set-up shifts). Then a probability density-based 3D volume was extracted, the so-called Coverage Probability Volume, representing the per-voxel probability of encountering the bladder in the 3D space across treatment time. In the case where the bladder was identified at a specific voxel location across all 12 CBCT timepoints and the planning CT, the voxel was assigned a 100 % coverage probability. Conversely, if the bladder appeared in e.g. only 5 out of the 12 CBCTs and the planning CT, the coverage probability for that voxel was approximately 38 % (5 out of 13). This Coverage Probability Volume based on data from the entire RT was used as the reference to compare with the outcome of the predictive models (Suppl. Mat. C).

**Fig. 1:**
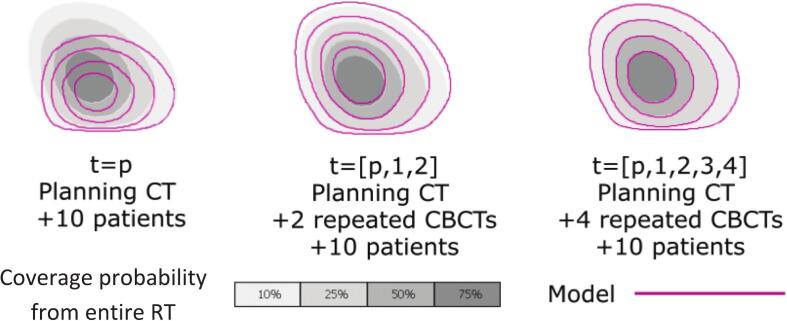
Schematic representation of the Coverage Probability Volume (gray) and the 10, 25, 50, and 75 % coverage probability isocontours for a given patient (magenta).

To evaluate the convergence of the models, the Dice Coefficients between the Coverage Probability Volume from the generated models and Coverage Probability Volume from the entire RT course were calculated. The Dice Coefficients for the three predictive models were evaluated at coverage probability isocontours of 10 %, 25 %, 50 % and 75 % (Fig. 1). These probabilities aligned with significant levels within the interquartile range, although other isovalues could have been examined as well. Further details of the methodology were described by Furmanova et al [20].

### 2.4 Statistical analysis

Initially, it was investigated whether the planning CT and the first four CBCTs could provide indications of the main modes of anatomical variability in the bladder clusters (i.e., patient groups with distinct anatomies), which were transferable to all CBCTs. Second, the 17 shape descriptors were assessed for statistically significant differences with respect to differentiation between bladder clusters (i.e., descriptors indicative of patient groups with distinct anatomies). Third, it was assessed if any of the 17 descriptors could be used for differentiating between patients with and without GU late ≥Grade 2 effects. Finally, it was evaluated whether the planning CT and the first four CBCTs were sufficient to reliably predict anatomical variability, and potentially be used to classify patients into a particular cluster. The first three tests were performed in the descriptive analysis (Section 2.2) while the fourth was tested in the generation of predictive models (Section 2.3).

## 3 Results

**Fig. 2:**
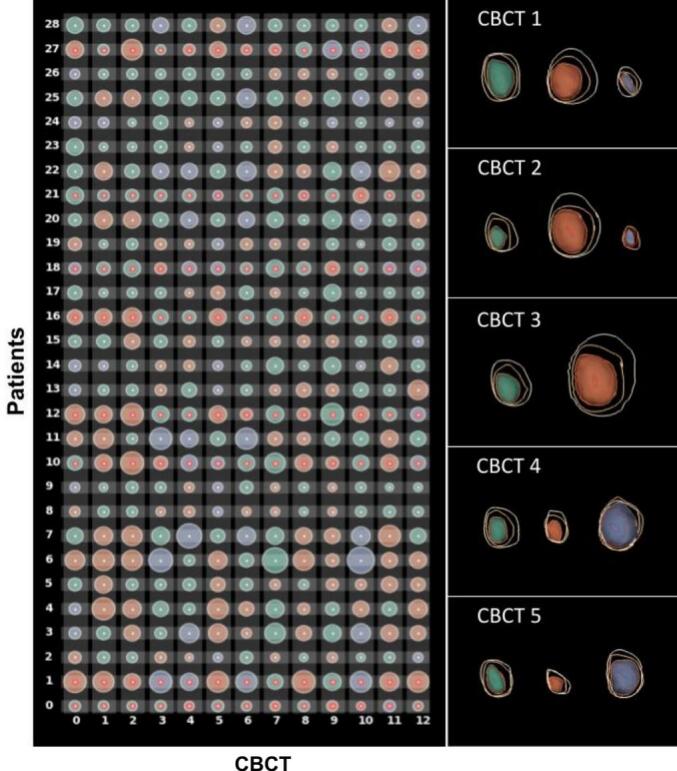
Contingency matrix (left) representing bladder volume changes and cluster assignment for all patients (rows), for all CBCTs (columns). Here, each bladder has been abstracted to a circular glyph: the color indicates the cluster assignment (Cluster 1 – orange, Cluster 2 – green, or Outliers – purple), the size indicates the volume of the bladder, and red dots indicate presence of GU ≥Grade late effects. Example of the three patient clusters (Right) computed for the first four CBCTs, where color indicates cluster assignment and the contours indicate the coverage levels (100 %, 75 %, 50 %, 25 %, 10 % going outwards from the center of the bladder). The 50 % coverage level is additionally indicated by a 3D surface rendering of the bladder (i.e., the colored green, orange, or purple surface).

Using the bladder contours from the planning CT and the first four CBCTs, the bladder volumes could be classified into two main clusters (Fig. 2) with distinct shape characteristics and comprising 84 % of the total number of bladders. One cluster contained contours with smaller bladder volumes, while the other cluster contained larger bladder volumes. The remaining 16 % was grouped into a third cluster (Fig. 2) that comprised the outliers (patients that could not be classified into any group). The cluster containing smaller bladder volumes, was significantly different from the other cluster and the outliers (*p* < 0.01). Cluster assignment and differences in bladder volumes remained when data from the entire RT course (12 CBCTs) were pooled in the t-SNE/Gaussian Mean Shift pipeline (again 84 % of the bladder contours were included in the two main clusters).

Significant differences between cases and controls were observed at each cluster for seven out of the 17 descriptors: convexity (i.e., measure of curvature) and elliptic variance (i.e., similarity to an elliptical shape) along the three principal axes, and compactness (i.e., sphericity).

In the cluster containing the small bladder volumes, the bladder volumes with more convex and round shapes were associated with higher risk of GU late effects ≥Grade 2. In the other cluster containing large bladder volumes, and opposite to the cluster of small bladder volumes, the bladder volumes with more concave and elliptical shapes were associated with a higher risk of GU late effects ≥Grade 2. These significant differences in shape descriptors between patients experiencing and not experiencing late effects, both across and within clusters, were also observed from the analysis including data of all 12 CBCTs (Suppl. Mat. B).

Within each cluster, the convexity measure along the craniocaudal axis, the elliptic variance along the transverse axis, and the compactness were significantly different between patients with and without late effects (*p* < 0.05). Across clusters the convexity along the longitudinal and the sagittal axes, the elliptic variance along both frontal and sagittal axes, and the compactness were significantly different between patients with and without late effects (ANOVA test, p < 0.05). Overall, low to medium correlation was observed among the 17 descriptors. The same results were observed from the analysis including data from the first four and all 12 CBCTs. In general, patients with late effects presented smaller convexity (i.e., they were less convex) in the AP direction, higher elliptic variance in the LR direction (i.e., they were less close to an elliptic shape), and bigger compactness (i.e., more spherical) than patients without late effects.

Finally, the goodness of the predictive models was evaluated by the Dice Coefficients between the predictive models and the Coverage Probability Volumes from the entire treatment course. The Dice Coefficients (median ± interquartile range) for the predictive model against different levels of Coverage Probability Volumes were: at the 10 % isocontour of Coverage Probability Volume 92 ± 10 % for planning CT only, 95 ± 3 % for planning CT and first two CBCTs, and 96 ± 4 % for planning CT and first four CBCTs; while for the 75 % isocontour of Coverage Probability Volume 88 ± 13 % for the planning CT only, 92 ± 5 % for planning CT and first two CBCTs, and CBCTs 93 ± 6 % for planning CT and first four CBCTs.

## 4 Discussion

In this study, by using a previously developed visualization tool to analyze bladder shape descriptors [20,21], it was shown that i) patient clustering remained identical when using the first four CBCTs of the first week and the 12 CBCTs acquired during the entire treatment course; ii) that convexity, elliptic variance and compactness were significantly different between patients with and without late effects (p < 0.05); and ultimately, iii) that the predictive models based on the planning CT and the first four CBCTs showed a good agreement (Dice Coeffs. > 93 %) compared to voxels' probability of belonging to the bladder.

Current planning protocols for radiotherapy of prostate cancer include planning dose-volume objectives to keep risk of developing late effects within a clinically acceptable range [22,23]. This late effect risk assessment is however based on the planning CT, which is a single instance of patients’ anatomy. In a previous study using the same set of patients, it was demonstrated that strict adherence to image-based assessment bladder filling protocol might reduce the risk of developing GU late effects, and despite significant variability in bladder volume there were no significant differences between planned and delivered dose distributions [11]. On the other hand, late effect risk assessment based on dose-volume metrics disregards anatomical locations of voxels receiving a particular dose (and in the setting of bladder often scored to the relative volume). These metrics are also blind to patient-to-patient variation in organ at risk size, shape, and their behavior through the treatment course. In other words, conventional dose-volume based approaches to assess the risk of late effects do not allow for patient stratification based on anatomical features and their day-to-day variations [24]. Additionally, the risk of late effects is typically evaluated under the umbrella of a certain grade of late GU effect (e.g. RTOG ≥ Grade 2), disregarding the wide range of symptoms that might be present (urgency, pain, leakage, etc.). These approaches are however needed to increase the statistical power, given the low number of patients presenting each symptom, but it is indeed a limitation in deciphering accurate dose–response relationships. This study showed that geometry-based measures of the bladder might be used to classify patients’ anatomy, and were associated with the risk of developing GU late effects.

The main objective of using image-guidance in RT of prostate cancer is to ensure correct positioning of the target volume, with emphasis on the prostate/rectum interface. Gross deviations from the planning CT in size/filling of organs at risk, e.g., gas in rectum, or substantially insufficient bladder filling, will prompt taking the patient off the couch and asking to take action to remedy the situation. However, moderate deviations are acceptable. These strict protocols minimize both dose-volume metrics and anatomical differences between planned and during treatment, keeping delivered dose-volume metrics within acceptable levels [11]. A previous work using this set of patients showed that accumulated treatment dose-volume metrics did not show higher predictive power for risk of developing late effects compared to dose-volume metrics extracted at the planning stage. But on the contrary, the change in bladder volume from planning CT to during treatment CBCT was significantly different between cases and controls [12]. In addition, the spatial distribution of dose within the bladder has been shown to play a key role in the development of different side effects after treatment [13,14,25]. In the same direction of these findings, the present study showed that overall bladder anatomy changes (84 % of the patients) occurring in the first week of treatment were representative of the entire treatment.

Predictive models, compared to previous approaches based on dose-volume metrics, might allow updating patient-specific risk assessment during the treatment course and open the way for a potential early adaptation of the treatment. This tool has shown high predictive power for risk of late effects due to anatomical variability, based on bladder shape and position descriptors from the first week of treatment. This will enable us to establish appropriate action levels at a very early stage of the treatment course to adapt dose delivered to organs at risk and adhere to target coverage [26]. In the setting of cervical cancer [27], or intra-fractional changes in RT for prostate cancer [28], valid models predicting organ position have already been found.

The present study has been carried out using a matched case-control cohort of patients, of a modest no. of patients. Further analysis and validation using larger cohorts are needed. On the other hand, the evaluation of fraction-wise late effect risks requires contouring on daily imaging, increasing operational time; although new auto-contouring methods might speed up the process and reduce human intervention. The study also used patient data for a fractionation schedule of 43–45 fractions, but this methodology can be readily translated to other fractionation schedules, including moderately fractioned ones, since risk of late effects is dependent on (complex) geometry. Using this approach towards stereotactic and/or hypo-fractionated treatments is challenging as by the time the needed data are collected and processed, treatment is completed. Nevertheless, this approach is potentially useful to stratify patients according to the risk groups for follow-up.

In conclusion, this study showed that changes in bladder shape descriptors during the first week of treatment were representative of changes during the entire treatment course. Changes in bladder shape descriptors may be used to differentiate between patient groups, and between patients presenting and not presenting late effects within each group. Bladder shape descriptors extracted from the first week of treatment might be used as early predictors of the risk of developing GU late effects following radiotherapy of prostate cancer.

## Declaration of competing interest

The authors declare that they have no known competing financial interests or personal relationships that could have appeared to influence the work reported in this paper. Given his role as Editor-in-Chief, Ludvig Muren had no involvement in the peer review of this article and had no access to information regarding its peer review. Full responsibility for the editorial process for this article was delegated to another journal Editor. In addition, Vitali Moiseenko is an Editorial Board Member for this journal and was not involved in the editorial review or the decision to publish this article.
