# Supplementary material for: Risk of genitourinary late effects after radiotherapy for prostate cancer associated with early changes in bladder shape
Source: Phys Imaging Radiat Oncol. 2025 Oct 31;36:100855. doi: 10.1016/j.phro.2025.100855 (PMC12603767; doi:10.1016/j.phro.2025.100855)
Supplement: Supplementary Data 1 [file mmc1.docx]

**Supplementary material**

1. Bladder shape descriptors extracted at each CBCT and planning CT

The shape descriptors of the bladders are calculated based on Peura and Livarinen [16], together with the additional volume of the organs:

- **C** = {Cx, Cy, Cz}: the coordinates of the center of mass on the 3 main anatomical planes. This position is referred to as the center of the bladder volume.
- **L** = {L1, L2, L3}: the three main principal axes in the form of eigenvalues (referred to as Principal Axes in [16]).
- **Conv** = {Convx, Convy, Convz}: the convexity on the 3 main anatomical planes, defined as the ratio between the perimeters of the minimal convex covering of an object, and the object perimeter [16].
- **A** = {Ax, Ay, Az}: the areas at the intersections with the 3 main anatomical planes. Defined as the proportional mean-squared error with respect to solid circle (referred to as Variance in [16]). Being zero for a perfect circle and increasing with shape complexity and elongation.
- **Evar** = {Evarx, Evary, Evarz}: the elliptical variances on the 3 main anatomical planes. By fitting to ellipse each projection of the bladder in the 3 anatomical planes and measuring the mapping difference between the object and the fitted ellipse [16].
- Compactness: how close to a sphere an object is, defined as the ratio of the squared perimeter and the area of an object [16]. Being the minimum 2π in a circular shape, and approaching infinity in thin/complex shapes.
- Volume: volume of the bladder.

Then for each patient and at each CBCT a 17-D vector is extracted with all the above calculated metrics. The 17-D vectors containing the shape descriptors are then:

- normalized to have the same units (e.g., volume is in mm^3^ and others are without unit).
- standardized according to the standard score.

1. Dimensionality reduction and clustering within the descriptive analysis

Then, the 17-D vectors are fed to a t-distributed Stochastic Neighborhood Embedding ([t-SNE) algorithm](http://www.jmlr.org/papers/volume9/vandermaaten08a/vandermaaten08a.pdf) for dimensionality reduction into 2D. The output is fed to a [mean-shift clustering algorithm](http://www.comaniciu.net/Papers/MsRobustApproach.pdf) to determine which patients are similar to each other. The dimensionality reduction part is necessary to avoid computational load (calculating similarities in 2D instead of 17-D is less time-consuming, and takes a few seconds) and the curse of dimensionality. Clustering is done based on the first CBCT and cluster matching is performed to ensure correspondence. The outcome of the clustering is a cluster assignment for each patient at each CBCT (i.e., 1, 2, or 3) indicating that a patient at one specific CBCT belongs, e.g. to cluster 2. On the outcomes, we do an ANOVA to examine whether all group means differ from one another, and then t-tests to compare pairwise groups to see which pairs differ from each other.

*Suppl. Material - Table 1: Average bladder volume and standard deviation, and late effects ratios for each cluster using the planning CT and the first four CBCTs.*

|  | Cluster 1 (49%) | Cluster 2 (35%) | Outliers (16%) |
| --- | --- | --- | --- |
| Volume Mean (mm^3^) | 155851 | 239691 | 240460 |
| Volume SD (mm^3^) | 126344 | 130936 | 142524 |
| No-Late Effects (N) | 53 | 34 | 18 |
| Late Effects (N) | 18 | 17 | 5 |
| Ratio Late Effects | 0.34 | 0.50 | 0.28 |

*Supp. Material - Table 2: Average bladder volume and standard deviation, and late effects ratios for each cluster using the planning CT and the twelve CBCTs.*

|  | Cluster 1 (46%) | Cluster 2 (38%) | Outliers (17%) |
| --- | --- | --- | --- |
| Volume Mean (mm^3^) | 167028 | 215841 | 223121 |
| Volume SD (mm^3^) | 76436 | 110783 | 141342 |
| No-Late Effects (N) | 128 | 99 | 45 |
| Late Effects (N) | 45 | 42 | 17 |
| Ratio Late Effects | 0.35 | 0.42 | 0.38 |

Additionally, since the clustering was based on the outcome of the t-SNE where each bladder contour was represented in a 2D vector, there was no need to perform a multiple comparison correction (e.g. Bonferroni correctly). Following, the ANOVA test was performed including all descriptors both across and within clusters. Performing the ANOVA test using the actual shape descriptors allowed to link results with metrics directly extracted from the contours.

1. Predictive analysis

Bladder volumes contoured for each patient, and at each CBCT were transformed into a unified volumetric representation by creating binary masks, and registering CBCTs at the treatment isocenter. Then a probability density-based shape descriptor was extracted, including the so-called Coverage Probability Volume, representing the per-voxel probability of encountering a given organ in the 3D space.

Based on the density-shape descriptor, each patient was matched with the 10 most similar patients to be used in predictive analysis. The matching was done employing the three organs: bladder, prostate and rectum at each CBCT.  For each patient, a mean shape descriptor was computed along with deformations with respect to the mean shape at individual CBCT. Using these deformations a generative Principal Component Analysis (PCA)-based model was established and sampled. Predicted Coverage Probability Volumes were computed from 1000 samples. For each patient three models were computed: (Model 1) contours from the planning CT (only the first timestep), (Model 2) contours from the planning CT and the two first CBCTs (three timesteps), and (Model 3) contours from the planning CT and the first four CBCTs (five timesteps). See Suppl. Material - Fig. 2.


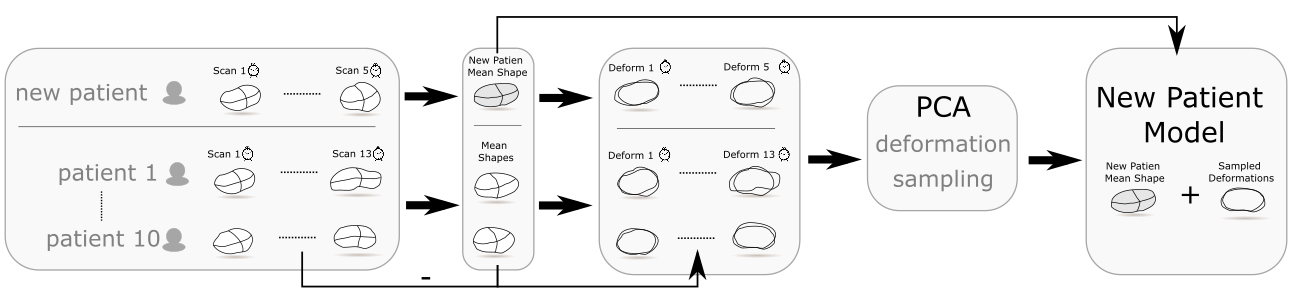


*Supp. Material - Fig. 2. Schematic representation of the predictive analysis based on the density-shape descriptor models.*

The convergence of the models was evaluated using Dice Coefficient of the predicted Coverage Probability Volume versus Coverage Probability Volume from the entire RT course, and for a given patient at volume isocontours corresponding to 10, 25, 50, and 75% coverage probability (Supp. Material - Fig. 3).


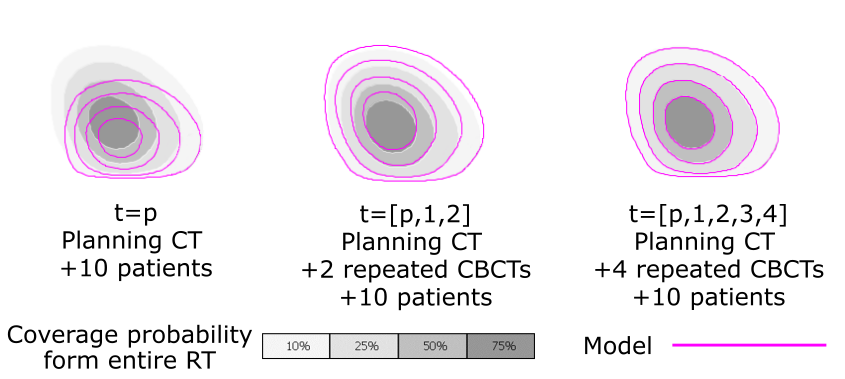

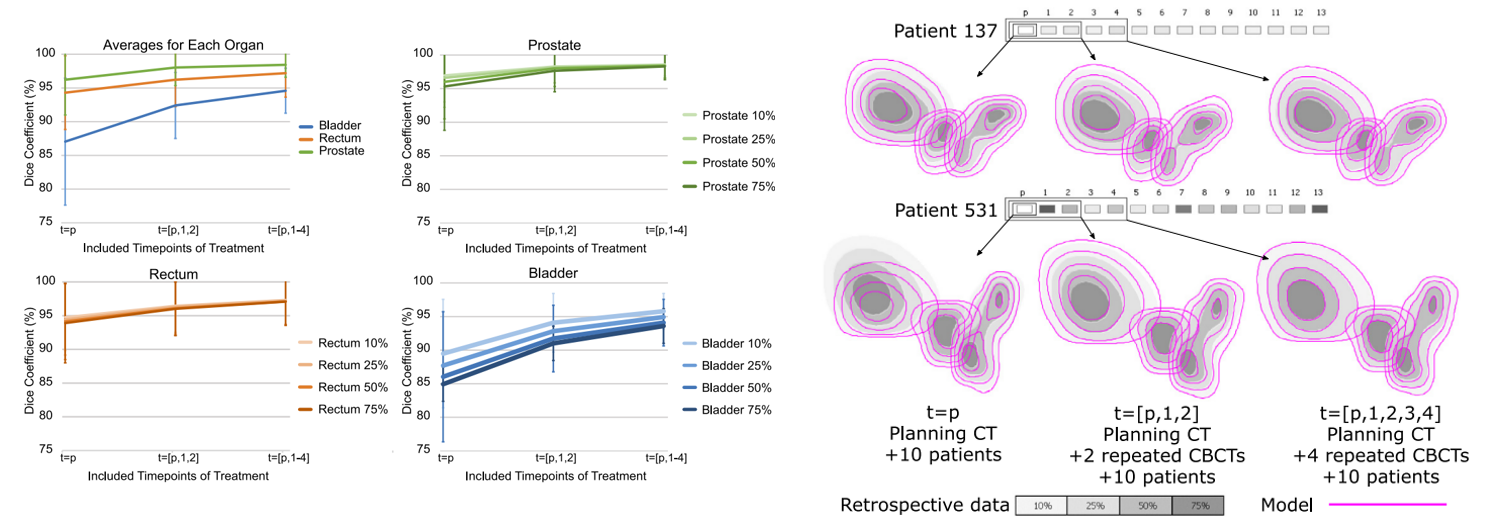


*Supp. Material - Fig. 3. Left: Schematic representation of the Coverage Probability Volume (gray) and the 10, 25, 50, and 75% coverage probability isocontours for a given patient (magenta). Right: Convergence of the predictive analysis for just the planning CT, the planning CT + two CBCTs, and the planning CT + four CBCTs. With the planning CT + four CBCTs, the model converges for 93±5.5% of the cases.*
